# High-quality genome assembly of *Chironomus riparius* and its population history in European populations

**DOI:** 10.1093/g3journal/jkaf189

**Published:** 2025-10-17

**Authors:** Laura C Pettrich, Robert King, Linda M Field, Ann-Marie Waldvogel

**Affiliations:** Institute of Zoology, Department of Biology, Universitat zu Koln, Zülpicher Straße 47b, Cologne 50674, Germany; Rothamsted Research, West Common, Harpenden AL5 2JQ, United Kingdom; Rothamsted Research, West Common, Harpenden AL5 2JQ, United Kingdom; Institute of Zoology, Department of Biology, Universitat zu Koln, Zülpicher Straße 47b, Cologne 50674, Germany; Limnological Research Station, School of Life Sciences, Technical University of Munich, Hofmark 1-3, Iffeldorf 82393, Germany

**Keywords:** *Chironomus riparius*, chromosome-scale genome assembly, long-read sequencing, population genomics, demographic inference

## Abstract

The aquatic midge *Chironomus riparius* is an established indicator taxon for the assessment of water quality as of the European Water Framework Directive. Here, we present a novel long-read genome assembly generated with PacBio HiFi and Hi-C sequencing, which achieves chromosome-scale resolution with an assembly size of 192 Mb, an N50 of 59 Mb, and a BUSCO completeness of 99.0%. Four chromosomes with their predicted centromeric regions and 10 unplaced scaffolds were assembled containing 15,439 protein-coding genes. Chromosome-level resolution in nonmodel species is often limited, posing challenges for population genomic studies that depend on high-quality reference genomes. Reanalyzing genomic data of natural *C. riparius* populations, we demonstrate the improved accuracy of population genomic estimators based on the high-quality reference genome. The high contiguity and completeness of the assembly enhanced demographic inference with Sequential Markovian Coalescent (MSMC2) modeling. Our results suggest that population divergence began in an ancestral lineage during the late Pleistocene to early Holocene, consistent with paleoclimate records from Central Europe.

## Introduction

Understanding the evolutionary history of natural populations is essential for uncovering how species respond to environmental change over time. Advances in genomic technologies have enabled increasingly accurate reconstructions of past population dynamics, offering new insights into evolutionary and ecological processes.

The aquatic midge *Chironomus riparius*, commonly known as the harlequin fly, is widely distributed across the Holarctic (Armitage et al. 1995) and is an established indicator taxon of the saprobic index to assess water quality as implemented in the European Water Framework Directive. Furthermore, the species is an emerging model system in molecular genetics (Schmidt 1981, 1984; Hägele 1984; Hankeln and Schmidt 1987; Bovero et al. 2002) and population genomics research (Oppold et al. 2017; Waldvogel et al. 2018). Early studies using polytene chromosomes characterized its genome structure, heterochromatic banding patterns, and chromosomal integrity in hybridization experiments with related *Chironomus* species (Schmidt 1981; Hägele 1984; Hankeln and Schmidt 1987; Bovero et al. 2002). These efforts laid the foundation for further molecular genetic research, employing various methodologies and sequencing strategies (Oppold et al. 2017; Schmidt et al. 2020; Schreiber and Pfenninger 2021). The *C. riparius* genome project highlights advancements in sequencing technology and assembly strategies (Oppold et al. 2017; Schmidt et al. 2020). However, previous population genomic studies, like demographic analyses, relied on a fragmented genome draft (Waldvogel et al. 2018). Here, we present a novel high-quality genome assembly for the species, resolving chromosome-scale details, and marking a milestone for future research with this potential model organism. Utilizing PacBio HiFi sequencing for high-accuracy long reads combined with Hi-C, a proximity-ligation scaffolding method, resulted in the final assembly.

Genomic data provide key insights into patterns of population growth, decline (Li and Durbin 2011; Schiffels and Durbin 2014), and admixture (Luikart et al. 2003). Studying the demographic evolution of natural populations is facilitated by whole-genome resequencing data of multiple individuals (Waldvogel et al. 2020; Bourgeois and Warren 2021). Sequential Markovian coalescent models (SMC models) (Li and Durbin 2011; Wilton et al. 2015; Schiffels and Wang 2020) trace back mutation and recombination events to infer population demography, hence interpreting patterns along the genomic sequence. The accuracy of the demographic inference might ultimately depend on the resolution of these patterns, which can be shaped by various factors such as mutational and recombination landscape and transposable elements (TEs), microsatellites, and DNA methylation (Sellinger et al. 2023). Comparing our new demographic estimates with those from Waldvogel et al. (2018) offers insights into the significance of reference genome resolution for SMC studies. We estimate population demography using the Multiple Markovian Coalescent (MSMC2) model, which applies a hidden Markov model to estimate genealogies (Schiffels and Wang 2020). The chromosome resolution of the assembly improved MSMC2 preconditions, i.e. restricting the analysis to scaffolds with a minimum length of 500 kb (Schiffels and Wang 2020). The inference of the recent past is known to reach better resolution when more sequences are investigated (Schiffels and Wang 2020). Under the assumption that young haplotype blocks should have larger sizes, the improved genome quality additionally contributes to the resolution of the more recent population history (Stumpf and McVean 2003). The likelihood of unmapped reads is higher in assemblies based on short-read sequencing which means that if a fragmented genome is not of high resolution in problematic regions, estimates of population history will be biased (Sellinger et al. 2021). Technical errors, like spurious SNP calling or incorrect detection of TEs, showed to decrease the accuracy of population history estimates (Sellinger et al. 2021). These errors are more likely, and more difficult to control for, in fragmented genomes with low resolution of low complexity regions. To explore how the enhanced resolution of the genome assembly affects the inference of population history, we hypothesize that the increased resolution of population genomic estimators will significantly improve the accuracy of demographic inferences. Furthermore, we integrate our genomic estimates with paleoclimate data (Karger et al. 2020, 2023) to explore how past environmental changes potentially shaped population history.

## Materials and methods

### Sample origin and sequencing

The reference genome of *C. riparius* provided by Rothamsted Research (West Common, Harpenden, United Kingdom) was assembled from a single female individual of a long-term laboratory strain (German origin, live material received from Syngenta, procured by the Innovative Environmental services [IES] Ltd, Switzerland, no aniso-female line). DNA was extracted using the MagAttract HMW DNA Kit (Cat. no. 67563, QIAGEN, Hilden, Germany). For PacBio HiFi sequencing, 450 ng of high molecular weight DNA was sequenced using 1 SMRT Cell 8 M on the PacBio Sequel II system, generating ∼30 Gb of HiFi data. Hi-C libraries were prepared using the Arima-HiC Kit (Arima Genomics, San Diego, CA, United States) by Arima Genomics following the manufacturer's 6-h protocol and sequenced on an Illumina platform using 150 bp paired-end reads yielding ∼926 million ready and 723X coverage. Whole-genome resequencing data of 5 natural *C. riparius* populations, previously investigated in Waldvogel et al. (2018), were used to assess the improved accuracy of population genomic estimators on the novel assembly. The origin of the different populations was from Rhône-Alpes (MF) and Lorraine (NMF) in France, Hesse in Germany (MG), Piedmont in Italy (SI), and Andalusia in Spain (SS). The data, trimmed resequencing datasets of 4 individuals per 5 populations respectively, were downloaded from the European Nucleotide Archive (ENA: 150-bp paired-end, Illumina sequencing data; Project number PRJEB24868).

### Genome assembly and annotation

**Fig. 1.**
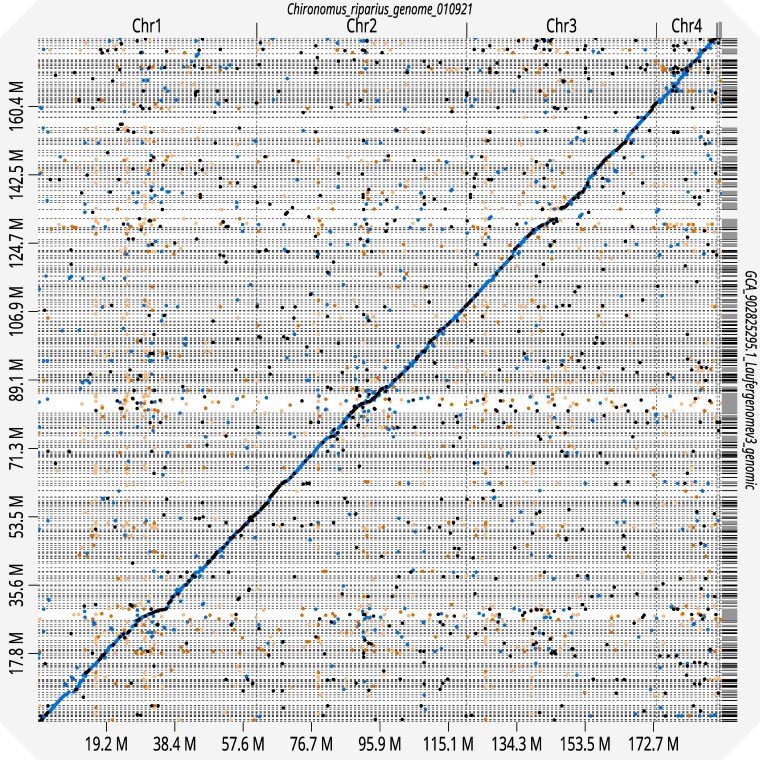
Dot plot comparing old genome assembly (752 scaffolds) (Schmidt et al. 2020) to new assembly (4 chromosomes and 10 unplaced scaffolds) created using D-GENIES (Cabanettes and Klopp 2018). Black dots show an identity of >75%, blue indicates <75% identity, orange <50% identity, and light orange <25% identity.

The genome contains 4 chromosomes and 10 scaffolds with a size of 192 Mb (NCBI accession number: PRJEB47883). The initial assembly was performed using Hifiasm (Cheng et al. 2021, 2022) to assemble the PacBio HiFi data into primary contigs. For chromosome-level scaffolding, the Hi-C data were processed with Juicer (Durand et al. 2016) to generate contact matrices, followed by 3D-DNA (Dudchenko et al. 2017) to perform automated scaffolding and identify potential misassemblies. Manual curation was performed using Juicebox (Durand et al. 2016) to inspect Hi-C contact maps, correct misassemblies, and optimize scaffold ordering and orientation. Following manual curation, Juicer was rerun to validate the final assembly quality and confirm proper chromosome-scale scaffolding. Haplotigs were identified and removed using purge_haplotigs (Roach et al. 2018). Unmapped reads were mapped back to the original assembly to check for missing sequences and incorporated into the final assembly. The Hi-C contact maps confirmed proper chromosome-scale assembly with clear diagonal signals and absence of misassembly artifacts, validating the quality of the final genome assembly. To assess the general quality of the genome assembly, the software Blobtoolskits (v2.6.5, Challis et al. 2020) was utilized and an analysis for BUSCO (v5.3.2) completeness was performed using the insecta_odb10 dataset together with the Augustus gene predictor (v3.5.0) in a long run (Simão et al. 2015; Manni et al. 2021a, 2021b). The web application D-GENIES (Cabanettes and Klopp 2018) was utilized (aligner: Minimap2 v2.28, options: Many repeats) to compare the new assembly to the previous version (Schmidt et al. 2020) which resulted in a dot plot of the alignment (Fig. 1) and a summary plot (Supplementary Fig. 3).

An RNA-seq transcriptome was assembled from public data (BUSCO Insecta: C: 94.7% [S: 53.7%, D: 41.0%], F: 0.4%, M: 4.9%) and used in the Maker2 (Holt and Yandell 2011) annotation pipeline with trained Augustus (Stanke et al. 2008) and Genemark (Borodovsky and McIninch 1993) gene predictors. Data used included: PRJEB15223 (Larvae), PRJNA166085 (egg ropes, all 4 larval stages, pupae and male and female adults, larvae exposed to different concentrations of several model toxicants), PRJNA229141 (anterior and posterior early embryo), and PRJNA675286 (larvae—transition metal oxide exposure). PASA (Haas et al. 2003) was used to update the gene models to add UTR, correct existing models, and add isoforms. Non-coding RNA was annotated using Infernal (v1.1.4, Nawrocki and Eddy 2013).

A Pfam genomic track was created by converting to 6 reading frames and applying hmmer (Finn et al. 2015) to identify the loci of interest. Using this information, UDP, P450, ABC, and IRAC gene models were found and curated using mapped RNA-seq and a Maker gene annotation.

Two endosymbionts were assembled which included an unknown *Enterobacter* sp. (1,661,850 bp) and *Wolbachia* sp. (559,667 bp).

To identify the repeat content of the genome, sensitive soft masking of repeats on the genome was performed with RepeatMasker (v4.1.1, Smit et al. 2015) using a custom TE library by Vladimir Kapitanov which was modified by adding TE entries of Oppold et al. (2017). A cutoff score of masking repeats of 250 bp and the engine rmblast (-s -xsmall -cutoff 250 -u -gff -pa 10 -lib $LIB -dir $DIR -enginermblast $GENOME) was chosen for the RepeatMasker analysis.

### Prediction of centromere ranges

To predict centromere ranges, we used RepeatOBserver (Elphinstone et al. 2025), an R package that describes repeat patterns and predicts centromere location based on the repeat diversity. We used the standard settings for the analysis. In 2 separate runs, we created a AT DNAwalk (-g FALSE) and CG DNAwalk (-g TRUE). This tool estimates in which regions on each chromosome, the different repeat lengths have minimum abundance and outputs histograms summarizing the minimum abundance in 2 Mb windows. Additionally, a genomic form of the Shannon diversity index (*H*) is estimated in 500 kb windows. We plotted the Shannon diversity index with rolling mean over 100 windows using tidyverse (Wickham et al. 2019) and it was visualized with the addition of the R packages scales (Wickham and Seidel 2022), cowplot (Wilke 2024), patchwork (v1.1.3 Pedersen 2024), and zoo (v1.8–12, Zeileis and Grothendieck 2005).

### Processing of resequencing data

We mapped the Illumina reads of 5 European populations of *C. riparius*, from Hesse in Germany (MG), Rhône-Alpes (MF), and Lorraine (NMF) in France, Piemont in Italy (SI), and Andalusia in Spain (SS), to the novel high-quality reference genome assembly. Read quality was checked with FastQC (v0.11.9, Andrews 2010) and MultiQC (v1.12, Ewels et al. 2016). All preprocessing steps were performed according to Waldvogel et al. (2018). The trimmed reads were mapped separately to our reference genome using the tool bwa mem (-M -R’@RG\tID:$Population\tSM:$Individual\tPL:ILLUMINA’, v0.7.17, Li 2013). Low-quality alignments were removed using samtools (-q 30 -f 0 × 0002 -F 0 × 0004 -F0 × 0008, v1.13, Li et al. 2009) and to remove duplicates PicardTools (VALIDATIONSTRINGENCY SILENT -REMOVEDUPLICATES true, v2.26.10, Broad Institute 2018) was utilized. Mapping statistics were obtained using Qualimap (v2.2.2d, Okonechnikov et al. 2016) (Supplementary Table 3). Further details on all analysis steps can be found on GitHub: https://github.com/lpettrich/Crip_PopulationHistory_Centromere_2025.

### Variant calling

Variant calling and phasing were performed as suggested in the MSMC2 workflow (Schiffels 2016) for the 4 chromosomes (99.28% of the assembly). The unmasked reference genome was split by chromosome and mappability masks were created using SNPable (Li 2009), indicating mappable regions of the genome assembly. Following the standard workflow, variant calling was performed on the filtered bam files of the samples using bcftools (v1.13, Li 2011) and the script bamCaller.py supplied by msmc tools (Schiffels 2021) disregarding indels. This workflow generated VCF and mask files for each individual and chromosome which were necessary for generating the input files for MSMC2. Phasing was performed per chromosome utilizing SHAPEIT4 (v4.2, Delaneau et al. 2019). Since no reference panel for *C. riparius* was available, we merged all VCF files (bcftools merge) for phasing and separated them once again. To account for any missing information that is still contained in the unphased data, the phased and unphased VCF files were merged while keeping the unphased data and replacing it with phased data where it was available. All multiallelic SNPs were discarded and only biallelic sites were kept. Using the obtained masking files and variant calls, multihetsep files were generated using the script generatemultihetsep.py of the msmc tools. SNP densities were visualized in R (v4.2.1, R Core Team 2025) using R-Studio (v2022.02.0 + 433, RStudio Team 2025) together with several R packages, like tidyverse (v2.0.0, Wickham et al. 2019), scales (v1.2.1, Wickham and Seidel 2022), or cowplot (v1.1.1, Wilke 2024).

### Population genomic inference of demography

The Multiple Sequentially Markovian Coalescent (MSMC2) model was used to infer the population history of the 5 European populations (details on the input files in Supplementary Table 4). The generated multihetsep files were used for MSMC2 (v2.1.3, Schiffels and Wang 2020). Two populations were paired, resulting in a total of 16 haplotypes (4 diploid individuals per population) per dataset and a total of 10 different population pairs that were later analyzed in a cross-population analysis. The procedure of the cross-population analysis was to allow the first 2 MSMC2 runs that estimated the coalescence rate function within the population. Afterwards, an analysis across the populations was performed, selecting the population pairs. For the analysis, the used time segment pattern was 1*3 + 1*2 + 22*1 + 1*2 + 1*3 and ambiguous sites were skipped. In the end, the results were combined using the combineCrossCoal.py script from the msmc tools. Overall, the obtained output per population included time and population size estimates, as well as the relative cross-coalescence rate (rCCR) which is a measure indicating the divergence of populations. The rCCR ranges between 0 and 1 and every value above 1 is considered an artefact caused by the model. Time and population size estimates were averaged per population and then scaled to real time and effective population size. Time estimates were converted into generations by dividing it through the mutation rate of 4.27 × 10^−9^ (Waldvogel and Pfenninger 2021). By multiplying it with the generation time (Oppold et al. 2016; Waldvogel et al. 2018), these converted coalescence times were converted into years. The effective population size was obtained by inverting the coalescence rate and dividing it by 2 times the mutation rate. To only consider robust estimates, the first 5 entries as well as the last entry were excluded to account for uncertainties in the analysis caused by overfitting of the model. We estimated the time to the most recent common ancestor (tMRCA) of 1 population to validate the estimates of MSMC2. The mean haplotype length (MHL) was determined through the mean genome-wide heterozygosity and with regard to the switch error rate (SER). The SER of 2% in *Drosophila melanogaster* (Bukowicki et al. 2016) was used, the same as in the previous study of Waldvogel et al. (2018). The mean heterozygosity was determined from the ratio of diallelic SNPs per number of records. These values were needed to approximate the tMRCA of 1 population with the following formula: tMRCA = 1/(2 × *r* × MHL). The population recombination rate (*ρ*) (based on Schmidt et al. 2020) was approximated to the recombination rate in units of meiosis per generation (*r*) using this formula from Peñalba and Wolf (2020): *r* = *ρ*/(2*×c×N*_e_). In this context, *c* and *N*_e_ represented the organism's diploidy and effective population size, respectively. The effective population size (Oppold and Pfenninger 2017) was used for the calculation. To get an approximation of *r* in cM/Mb, the gene map length of *D. melanogaster* of 287.3 cM (Comeron et al. 2012) was used and compared with the gene map length of female *Clunios marinus* of 167.2 cM (Kaiser and Heckel 2012) because it is not yet available for *C. riparius*. The mean value of *r* of 1.36 cM/Mb was then used to calculate tMRCA (Supplementary Table 5). This value was compared with the one using the recombination rate of *D. melanogaster* of 2.1 cM/Mb (Mackay et al. 2012) which is the same value as used in Waldvogel et al. (2018). All plots were generated in R using tidyverse (Wickham et al. 2019) and the R packages egg (v0.4.5, Auguie 2019), RColorBewer (v1.1–3, Neuwirth 2022), and grid (v4.2.1, R Core Team 2025) (detailed list of all R packages in Supplementary Table 1). Plots were finalized using Inkscape (v1.3.2).

### Analysis of paleoclimate data

Results of the MSMC2 model of *C. riparius* were further compared with paleoclimate temperature data. Therefore, 22 bio1-datasets of the CHELSA-TraCE21k climate time-series were downloaded from CHELSA (Karger et al. 2020, 2023). Thus, the CHELSA-TraCE21k climate data provide information for the last 22,000 years before present (years BP) which referred to the Last Glacial Maximum (LGM). As such, contained the bio1-datsets annual mean temperatures and the here used 22 datasets (Supplementary Table 2) included timepoints from 1,000 years BP up to 22,000 years BP and were retrieved in steps of 1,000 years (i.e. millennial time-series).

Climate maps of Europe were created, and temperature data were extracted in R using the packages raster (v3.5–15, Hijmans and van Etten 2012) and maptools (v1.1–8, Bivand and Lewin-Koh 2023). A generalized linear model (GLMM) was generated using the R packages glmmTMB (Brooks et al. 2017), DHARMa (Hartig 2022), and broom.mixed (Bolker and Robinson 2024). Missing data points were interpolated which means more frequent values (rCCR) were interpolated to fit the less frequent (temperature) in 1,000-year intervals in the time range from 1,000 to 22,000 years ago. The rCCR was tested as response variable against temperature, time, and their interaction as the predictor variables and the populations as random effect with an added time-varying dispersion, using beta distribution and the BFGS algorithm for model optimization (glmmTMB(rel.cc ∼ temperature * time + (1 | Population), data = data, family = beta_family(), control = glmmTMBControl(optimizer = optim, optArgs = list(method = “BFGS”)), dispformula = ∼time). The model was fitted using 440 observations across 5 population groups. If the *P*-value was smaller than 0.05, it was considered significant. ChatGPT (OpenAI 2024) was utilized to improve scripts in R for statistical analysis and data visualization by either simplifying scripts with the creation of loops or for troubleshooting if the code was not working as intended. Suggestions from ChatGPT were reviewed and validated to ensure accuracy.

## Results and discussion

### Genome assembly at chromosome-scale resolution

The novel assembly of *C. riparius* resolves all 4 chromosomes with 10 remaining unplaced scaffolds. Genomes of 2 endosymbionts, *Enterobacter* sp. and *Wolbachia* sp., were additionally assembled from the metafraction of the data. We can show that the new assembly has a largely improved contiguity compared with the previous assembly from Schmidt et al. (2020). This is also shown by a dot plot comparing an alignment of these 2 assemblies using D-GENIES (Cabanettes and Klopp 2018) (Fig. 1). The D-GENIES summary showed that 74.90% shared an identity between 50 and 75%. The 13.33% had an identity higher than 75%. No match was found for 10.37% of the reference. Most of the sequences are matching, shown in a diagonal line, but the information in the new assembly is condensed. The new assembly consists of 14 scaffolds, with 4 representing chromosomes, compared with 752 scaffolds in the old assembly. Approximately 53 old scaffolds are merged into a single scaffold in the new assembly.

**Fig. 2.**
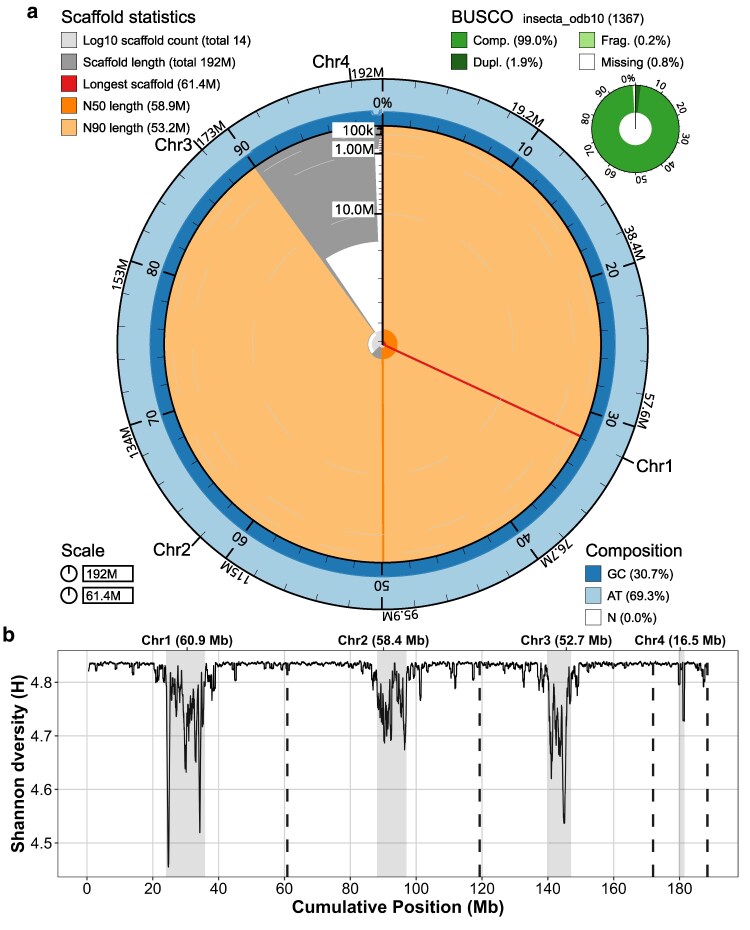
Summary on genome assembly and centromere regions. a) Snail plot summarizing the assembly statistics created with BlobToolKit. Scaffold statistics can be found on the top left. The longest scaffold (i.e. chromosome 1) is marked with a red line. The N50 value is marked with an orange line and the N90 value as a pale orange pie chart. Total genome length (Mb) is given, and each chromosome has been labeled at the end position using Inkscape (v1.3.2). The GC and AT composition are given in the outer circle in dark and light blue and the filling is proportionate to the percentage. The BUSCO analysis was performed against the insecta_odb10 database and values on completeness, fragmentation, duplication, and missing genes can be found on the top right. b) Plots of each chromosome show the rolling mean of Shannon diversity (*H*) for repeat length in 500 kb windows. If repeat content is less diverse *H* will decrease, the area with the lowest *H* is predicted to be centromere and centromere-flanking regions. Cumulative position (Mb) is given, but the end of each chromosome is marked by a dotted line.

The assembly spans 192 Mb with N50 of 59 Mb (Table 1, Fig. 2a). The assembly's completeness when compared with the single ortholog database of Insecta (insecta_odb10, *n* = 1367) scores 99.0% complete (97.1% single-copy and 1.9% duplicated), 0.2% fragmented, and 0.8% missing BUSCO genes. Overall, the assembly shows excellent continuity and completeness. Chromosome numbers were ranked with the descending length, with chromosome 1 being the longest scaffold of 61 Mb. The genome-wide GC content was estimated to be 30.7%. The assembly reveals a repeat content of 15.85%. In total, 15,439 protein-coding genes were annotated.

**Table 1.** Assembly statistics of the genome.

Assembly size	191,837,449 bp
N50	58,906,861 bp
GC content	30.7%
Repeat content	15.85%
No. of protein-coding genes	15,439
BUSCO (insecta_odb10)	C: 99.0% [S: 97.1%, D: 1.9%], F: 0.2%, M: 0.8%, n: 1367
Longest chromosome	61,357,614 bp
No. of chromosomes	4
No. of unplaced scaffolds	10

We applied RepeatOBserver to predict centromeric regions, following 2 approaches: Shannon diversity (*H*) of repeat lengths and histograms showing the abundance sum minima across 2 Mb windows. The histogram method estimates centromere positions at 33 Mb for chromosome 1, at 29 Mb for chromosome 2, at 25 Mb for chromosome 3, and at 9 Mb for chromosome 4. We assumed that low *H* values (<4.8) represent centromere-flanking regions, resulting in centromere ranges of 24–36 Mb for chromosome 1, 27–36 Mb for chromosome 2, 21–28 Mb for chromosome 3, and 7.7–9.5 Mb for chromosome 4 (Fig. 2b, Supplementary Table 7). The predicted centromere region of chromosome 4 differs as it is suggested to be located at the chromosome's end (Ilkova et al. 2007). The prediction of centromere regions on chromosome 4 may be influenced by the presence of Balbiani Rings (BR) or the nucleolar organizer regions (NOR), as both regions are known to contain extensive arrays of tandem repeats (Bäumlein et al. 1982; Wieslander 1994; Kutsenko et al. 2014; Gunderina et al. 2015), which could further contribute to this effect considering that centromeres are predicted based on repeat pattern.

### Population history compared with past climate history

**Fig. 3.**
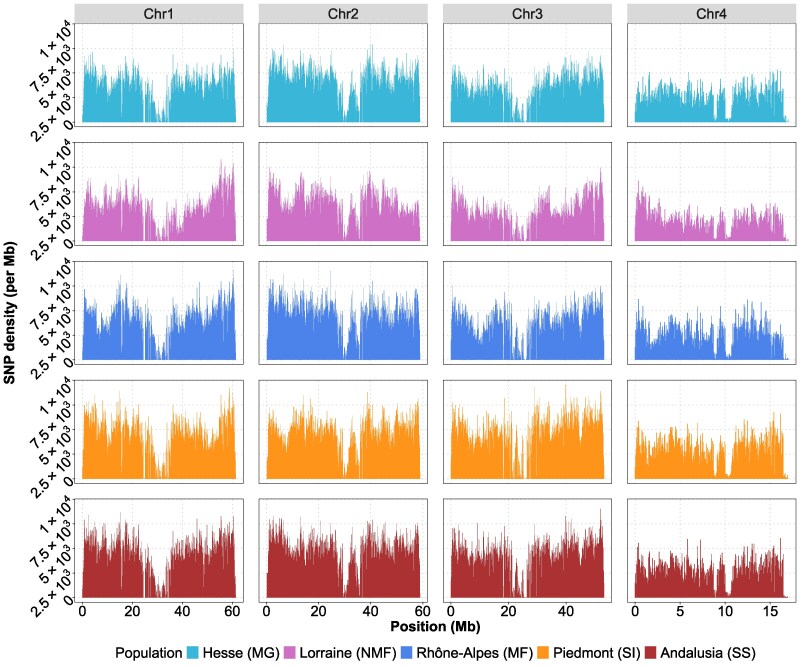
SNP density per chromosome and population of *C. riparius.* Based on combined multihetsep files of each population. Histograms are shown in 50 kb bins. Each segregating site divided by the bin size was counted to get SNP density.

SNP density was investigated for the direct input files of MSMC2 (i.e. multihetsep files) which include filtered biallelic SNPs (Fig. 3). There are certain regions with a decrease in SNP density which align with predicted centromere regions.

In the previous study, Waldvogel et al. (2018) used a fragmented genome assembly for an initial inference of population history in *C. riparius,* applying multiple sequential Markovian coalescence (MSMC2). Many short scaffolds and the lack of information about their placement hindered the inference of recombination sites and thus only 17.34% (30 scaffolds ≥ 100 kb) of the assembly were suitable for the analysis. With the new genome assembly, we were able to input 99.28% of the genome to the analysis—only excluding 10 unplaced scaffolds. The new assembly has a 99.0% BUSCO completeness (insecta_odb10), surpassing the 93.7% completeness of the old assembly (arthropod_odb).

**Fig. 4.**
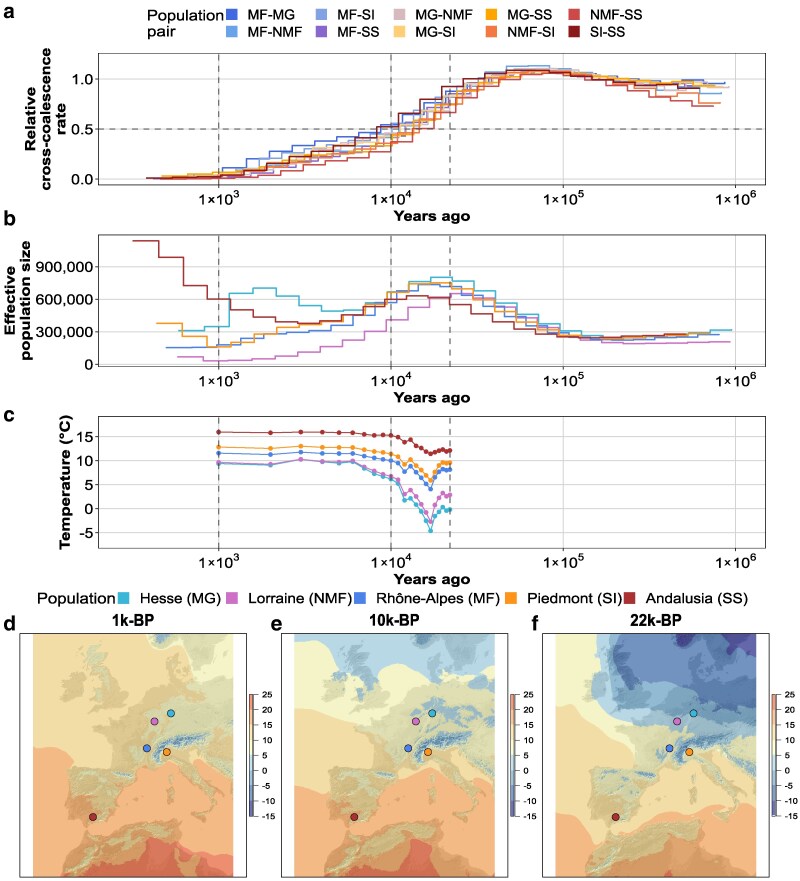
Comparison of the demographic history of *C. riparius* populations to available paleoclimate models. a) The rCCR of populations over the years reaching into the past estimated by the MSMC2 model. The population pairs are indicated by color, as shown in the legend on top. b) Inferred effective population size over years reaching into the past estimated by MSMC2. Color indicated by legend at the bottom. c) Annual mean temperature every thousand years (1k-BP–22k-BP) for the separate sampling locations. Dotted lines represent the time points of the maps. d–f) Maps of Europe showing the spatial temperature pattern across Europe 1k-BP, 10k-BP, and 22k-BP. Temperature (°C) is indicated by the gradient bar on the right. Dots refer to the sampling locations of the populations with the corresponding color code.

We assessed admixture between populations (Fig. 4a) and the history of effective population size (Fig. 4b) using the MSMC2 model. Separately from the MSMC2 run, we intended to validate the estimation robustness of the recent time horizon, by determining the tMRCA of the individuals per population by integrating a mean heterozygosity of 0.0083 and an informative MHL of 6,023 bases. The tMRCA was determined to be 10,468 generations when applying the mean recombination rate of 0.79 cM/Mb calculated from the genetic map length of *Clunio marinus* (Kaiser and Heckel 2012) or, alternatively, 6,092 generations for the mean recombination rate of *C. riparius* (1.36 cM/Mb; Schmidt et al. 2020) based on the genetic map length of *D. melanogaster* (Comeron et al. 2012). If we use the same recombination rate of *D. melanogaster* (2.1 cM/Mb, Mackay et al. 2012) as applied in the previous study of Waldvogel et al. (2018), we estimate a tMRCA of 3,953 generations. Despite the absence of a genetic map length for *C. riparius*, we get a good approximation for the time of the most recent common ancestor of the individuals per population. The true estimates will most likely lay closer to the first 2 estimates as the larger phylogenetic distance of Chironomidae to Drosophilidae (∼220 million years; Wiegmann et al. (2011)) could indicate substantial differences in genetic map length. Considering the species-specific recombination rate of the previous study of Schmidt et al. (2020), informative time intervals of the population history estimates of *C. riparius* range from ∼6,100 to 7,400,000 generations (Supplementary Fig. 1). Our findings highlight the significance of accurate genome assemblies, as the time ranges of demographic events were substantially shifted in the new analysis (Fig. 4ab). Waldvogel et al. (2018) estimated an informative time horizon between 150,000 and 351,000 generations in the past. To translate these values in years, the tMRCA in generations was multiplied with the mean generation time of the respective population (referring to estimates reported in Oppold et al. 2016) which resulted in a tMRCA of 609 to 1,046 years (Supplementary Table 5).

MSMC2 enables the analysis of admixture between populations by calculating the rCCR. We could observe a high relative rCCR in the ancient past (Fig. 4a) which is indicative for an ancient superpopulation. This admixture persisted until a peak 20,000 years ago (Fig. 4b). Toward the more recent past, the effective population size declined, leading to a dispersal of the population-specific estimates. A high admixture between populations can be found for our populations earlier than 10,000 years ago. Over time, the admixture decreased, and it is expected that the populations split into subpopulations once the rCCR fell below 0.5, ∼10,000 years ago. We compared the inferred population demography to paleoclimate models from the CHELSA database, to assess whether correlating temperature events could indicate a potential reason for the formation of different subpopulations (Fig. 4c–f). By reviewing the annual mean temperature of the population's habitat over time (Fig. 4c), a gradient can be found. Hesse and Lorraine are the coldest regions, followed by Rhône-Alpes and Piemont and Andalusia is the warmest region. Shifting the focus from only the population's habitat to the European region (Fig. 4d–f), a warming of all of Europe can be observed. The Last Glacial Maximum (LGM) happened 22,000 years before present (22k-BP) with mean temperatures dropping below 0 °C in many regions of Europe (Fig. 4f). In the latest period, around 1k-BP (Fig. 4d), most of the regions were warmer with only mountain ranges and the far North showing mean temperatures below 0 °C. The historical temperature estimates north of the alps indicate a shift from relatively cold temperatures (−15 °C to 5 °C) to moderate temperatures (0 °C to 15 °C). According to the model, southern Europe exhibited a consistently moderate climate (5 °C to 20 °C) starting from the earliest time point. The zenith of population sizes occurred prior to a temperature decline about 16,000 years ago. The lowest temperature, with an annual mean temperature of −4.6 °C, was registered 17,000 years ago at the site where the MG population is located today (Fig. 4c). Subsequently, temperatures increased, interrupted only by a minor decrease in temperature 12,000 years ago. The temperature increment slowed down from 7,000 to 1,000 years ago.

For the time range from 1,000 to 22,000 years ago, we tested whether there is a significant relationship between temperature, time, and rCCR in a GLMM using the beta family and BFGS optimization setting. We used the rCCR as response variable and tested the effects of temperature, time, as well as their interaction and the populations as random effect while allowing the dispersion to vary over time. The model revealed a significant effect (significant if *P* < 0.05) of time on the rCCR (*P* < 2e−16), while the interaction between temperature and time was also statistically significant (*P* = 0.00581). Temperature alone did not have a significant effect (*P* = 0.985). The model showed a significant increase in dispersion over time (*P* = 9.32e−7), indicating that variability in rCCR is not constant but grows as the analysis extends further into the past. The random intercept variance associated with population is relatively small, suggesting modest variability across populations (Supplementary Table 6 and Supplementary Fig. 2). As conclusion it appears that time had a bigger impact on the loss of admixture between populations.

### Biogeography supports population demography

Accurate population demography models allow us to interpret and correlate a population's history with the biogeographic history of its habitat. The temperature developments of the CHELSA traCE21k time-series dataset, covering the LGM up to 1,000 years before present (Karger et al. 2020, 2023), were compared with the geographical coordinates of the sampling sites of the 5 *C. riparius* populations (Fig. 4c and d). Correlation of these 2 very different data types allowed us to investigate whether paleoclimate models could have the potential to support sequence-based demographic estimations.

For our MSMC2 analysis, we adapted the time segment pattern, approximated the tMRCA of 1 population, and trimmed outer values to account for overestimations of the most recent and most ancient time interval, resulting from false positive or negative signatures of recombination (Schiffels and Wang 2020). We used the gene map length (Comeron et al. 2012) and SER (Bukowicki et al. 2016) of *D. melanogaster* to approximate the tMRCA, but it might be that results for tMRCA change once there is a gene map for *C. riparius* as we do not know the extent of the differences.

Our results suggest the origin of 1 ancestral population for the 5 investigated populations (Fig. 4a) as proposed (Waldvogel et al. 2018). Whilst the divergence of ancestral populations was previously proposed to have happened around 10,000 to 1,000 generations ago, our new estimates redefine this time frame. The admixture between the population shows a reduction between 500,000 and 10,000 generations ago and the rCCR reached a value of 0.5 at ∼100,000 generations in the past. When multiplying these coalescence estimates with the population-specific generation time available for this multivoltine insect species (based on Oppold et al. 2016), the estimates were converted into years. This conversion defines the period of divergence of the populations between late Pleistocene and early Holocene (Stroeven et al. 2016) (Fig. 4). The GLMM showed time as the major contributor of the decrease in admixture; however, temperature and time showed to have a significant interaction which indicates that it is difficult to interpret them independently from each other.

Starting from the LGM between 22,000 and 17,000 years ago, the ice margins in Europe started to recede which led to an almost ice-free central Europe 16,000 years ago (Ehlers 1990; Douda et al. 2014; Stroeven et al. 2016). Two major climate events can be found in the temperature data (Fig. 4c)—the Heinrich event (H1) around 16,800 years ago (Heinrich 1988; Bond et al. 1992; Hemming 2004) and the Younger Dryas around 12,000 years ago (Keigwin and Lehman 1994; Carlson 2010). Considering the biogeographic history of central Europe, it seems plausible that these climatic changes have contributed to the decreasing effective population size in the ancient population of *C. riparius*.

Interestingly, major extinction events happened globally in the late Pleistocene which have been partially linked to sudden climate change alongside major environmental shifts (Barnosky et al. 2004; Svenning et al. 2011; Kozyra et al. 2021). For ancient megafauna, these extinction events might have been accelerated by early influence of humans (*Homo sapiens*) (Varela et al. 2010; Bergman et al. 2023). Based on the size of our study system *C. riparius*, it seems unlikely that early anthropogenic impact influenced population decline. Climate change seems to be the more likely cause of a drop in population size for *C. riparius*. The aquatic larval stage is heavily dependent on water temperature. Changes in water temperature might have induced stress and diminished overall fitness; however, Foucault et al. (2018) demonstrated that its larvae can rapidly adapt to elevated temperatures, which may have been beneficial. Further, other ancient species also showed to tolerate shifts in their habitat as the realized ecological niche is not necessarily reflecting their actual fundamental niche (Tallavaara et al. 2015; Rey-Iglesia et al. 2021; Leonardi et al. 2022). It has been hypothesized that populations might have split from the central population in Rhônes-Alpes (MF) which is also in the center of the temperature ranges (Waldvogel et al. 2018). The increase in temperature in the late Pleistocene might have led to a first dispersal of the midges as more habitats became available after initial adaption (Oppold et al. 2016; Foucault et al. 2018). These newly dispersed populations showed more variability in their effective population sizes, especially the population from Spain (SS) and Hesse (MG). To disentangle the reasons for the variability is difficult as it is uncertain if the population always has been in this location or if its habitat has slowly migrated over time to its current location. There has likely been spatial variability in environmental conditions that we cannot cover, e.g. the temperature data from CHELSA is in 1000-year intervals with a 1-km resolution (Karger et al. 2023). As such, *C. riparius* depends on local waterbodies, and if drought events occur and waterbodies dry up, it could have negative impacts on its effective population size. If there was a lot of precipitation in certain areas, this could have been beneficial as there would have been many small waterbodies which could serve as breeding sites. In the Iberia region, it has been found that the early Holocene started dry but got more humid 10,000 to 9,000 years ago (Morellón et al. 2018) with the highest lake levels found from 8,100 to 5,700 years ago (Ilvonen et al. 2022), which does well align with what we found for the population from Andalusia (SS) as *N*_e_ started to increase in the mid of the Holocene when water levels were high (Fig. 4). However, they also found spatial and seasonal variations in climatic conditions and the climate became generally cooler and drier starting from 3,500 years ago (Fletcher and Zielhofer 2013; Liu et al. 2023). For the Hesse population (MG), we could document a peak in *N*_e_ for more recent times, the drop in *N*_e_ after the peak could be explained by cold winters (Fig. 4), as suspected for diatoms based on shorter lake mixing periods (Dreßler et al. 2011). Furthermore, records show that some glaciers in Norway began shrinking in the early phase of the mid-Holocene (around 5,000 years ago) (Bakke et al. 2005). This period was followed by a peak in the Hesse population, possibly indicating a more favorable climate for *C. riparius*. Subsequently, glacier growth occurred 2,220 years ago, suggesting a shift toward a less favorable climate (Bakke et al. 2005).

Both the potential expansion across an increased habitat space and the 2 cooling events are likely to have contributed to the decrease in the effective population size of the ancestral population. The decrease in the effective population size and potential dispersal (see also in Waldvogel et al. 2018) might have also led to a reduced admixture, finally leading to a split of 1 ancestral population into separate populations around 10,000 years ago. When comparing the population history of *C. riparius* with that of other European species, we observe, for example, that the effective population size of the bird species *Caprimulgus europaeus* expanded during warm periods and declined during cooler periods, with a rapid reduction during the LGM (Day et al. 2024). This pattern could also occur in other bird species feeding on chironomids, which could subsequently have affected the population size of *C. riparius*. The model species *D. melanogaster* is suspected to have dispersed from its sub-Saharan African origin and diverged ∼13,000 years ago (Kapopoulou et al. 2020). This timing is notably similar to the split of *C. riparius* populations, emphasizing a period marked by significant habitat shifts driven by environmental change between the Pleistocene and the Holocene (Hofreiter and Stewart 2009). As such, climate data could be used as an observational measure to support the demographic history estimation of *C. riparius* in Europe.

### More ancient population history estimates could be inferred for *C. riparius*

This increase in resolution on the demographic history of *C. riparius* in European populations can at least partially be explained by the high accuracy of PacBio long-reads leading to more accurate assemblies with better coverage and contiguity in low complexity and repetitive regions (Pollard et al. 2018), demonstrating the advantage of long-read sequencing coupled with Hi-C scaffolding (Guiglielmoni et al. 2022). However, some coalescence estimates of the model can also be explained by changes in population structure. We could clearly observe a split of populations over time in our MSCM2 analysis. A diverging population has many evolutionary consequences (Buffalo 2021) which can lead to overlaying signals that are difficult to distinguish by a coalescence model. In the investigated populations, we find a reduction in the effective population size (*N*_e_) alongside a reduction in the rCCR. However, the observed reduction in *N*_e_ could also be explained by other processes happening during the same time periods, for example, a change in the migration rates between migrating demes and the observed bottleneck could be explained by a shift in population structure (Nadachowska-Brzyska et al. 2022). There are also always recombination events that are not detectable, which means that the estimated population history is just an approximation (McVean and Cardin 2005). Linked selection can produce complex patterns along the genome which can concurrently influence estimates of *N*_e_, for example, a reduction in *N*_e_ in functional regions (Nadachowska-Brzyska et al. 2022). It has also been proposed that abundant species, like *C. riparius*, experience higher effects of linked selection causing a reduction in genetic diversity and recombination (Buffalo 2021). The more complete and chromosome-level assembly used resulted in more robust inferences of population history, spanning a wider period and reaching far deeper into the past. Our study also benefitted from the availability of a species-specific mutation rate estimate (Waldvogel and Pfenninger 2021) that additionally contributed to the increased resolution of population history in *C. riparius*.

## Summary

This study presents a novel genome assembly of chromosome resolution for the aquatic midge species *C. riparius*, an emerging model organism in experimental population genomics. We achieved improved population history estimates, providing a more accurate understanding of the demographic dynamics of the species. We could show a shift in the coalescence estimates using MSMC2 compared with the previous study and could match these new results with paleoclimate events. The increased resolution of the genome enabled the inference of a larger and more ancient informative time horizon.

Over and above these novel genomic insights into the genomic landscape of *C. riparius*, these genomic resources will be more generally valuable for comparative studies on insect genomics (Blackmon et al. 2017), experimental population genomics (Foucault et al. 2019), and chromosome evolution (Shaikhutdinov and Gusev 2022).

## Supplementary Material

jkaf189_Supplementary_Data

## Data Availability

The genome assembly can be downloaded at ENA (accession PRJEB47883). The Illumina sequences of the 5 populations were published under Waldvogel et al. (2018) and trimmed reads can be accessed at ENA (accession PRJEB24868). Scripts are available at the GitHub repository https://github.com/lpettrich/Crip_PopulationHistory_Centromere_2025. Input files necessary to run the scripts will be made available through Zenodo: https://doi.org/10.5281/zenodo.15177248. Supplemental material available at *G3* online.
