# Supplementary material for: High-quality genome assembly of Chironomus riparius and its population history in European populations
Source: G3 (Bethesda). 2025 Oct 17;15(12):jkaf189. doi: 10.1093/g3journal/jkaf189 (PMC12693489; doi:10.1093/g3journal/jkaf189)
Supplement: jkaf189_Supplementary_Data [file jkaf189_supplementary_data.docx]

## Supplementary Materials

Supplementary Table 1: List of tools and R packages used with citations.

|  | **Tools** | **Version** | **Citation** |
| --- | --- | --- | --- |
| **Software** | samtools | 1.13 | Li et al. (2009) |
|  | bcftools | 1.13 | Li et al. (2009) |
|  | bwa | 0.7.17 | Li (2013) |
|  | Trimmomatic | 0.39 | Bolger et al. (2014) |
|  | Picard Tools | 2.26.10 | Broad Institute (2018) |
|  | FastQC | 0.11.9 | Andrews (2010) |
|  | MultiQC | 1.12 | Ewels et al. (2016) |
|  | Qualimap | 2.2.2d | Okonechnikov et al. (2016) |
|  | bedtools | 2.31.0 | Quinlan and Hall (2010) |
|  | shapeit4 | 4.2 | Delaneau et al. (2019) |
|  | MSMC2 | 2.1.3 | Schiffels and Wang (2020) |
|  | msmc-tools | - | Schiffels (2021 Sep 7) |
|  | SNPable | - | Li (2009) |
|  | RepeatMasker | 4.1.1 | Smit et al. (2015) |
|  | RepeatOBserver | 1 | Elphinstone et al. (2025) |
|  | blastx | 2.12.0 | Camacho et al. (2009) |
|  | BUSCO | 5.3.2 | Manni et al. (2021), Simão et al. (2015) |
|  | BlobToolsKit | 2.6.5 | Challis et al. (2020) |
|  | D-GENIES | - | Cabanettes & Klopp (2018) |
|  | GNU parallel | 20160222 | Tange (2011) |
|  | Rstudio | 2022.02.0+433 | RStudio Team (2020) |
|  | R | 4.2.1 | R Core Team (2020) |
| **R packages** | ggplot2 | 3.3.6 | Wickham (2016) |
|  | dplyr | 1.1.4 | Wickham et al. (2023) |
|  | tidyverse | 2.0.0 | Wickham et al. (2019) |
|  | raster | 3.5-15 | Hijmans and van Etten (2012) |
|  | egg | 0.4.5 | Auguie (2019) |
|  | grid | 4.2.1 | R Core Team (2020) |
|  | patchwork | 1.1.3 | Pedersen (2024) |
|  | cowplot | 1.1.1 | Wilke (2024) |
|  | scales | 1.2.1 | Wickham and Seidel (2022) |
|  | gridExtra | 2.3 | Auguie (2017) |
|  | reshape2 | 1.4.4 | Wickham (2007) |
|  | RColorBrewer | 1.1-3 | Neuwirth (2022) |
|  | sf | 1.0-14 | Pebesma (2018), Pebesma and Bivand (2023) |
|  | rnaturalearth | 0.3.4 | Massicotte and South (2023) |
|  | naturalearthdata | 0.1.0 | South (2017) |
|  | rgeos | 0.6-4 | Bivand & Rundel (2023) |
|  | maptools | 1.1-8 | Bivand & Lewin-Koh (2023) |
|  | rgdal | 1.6-7 | Bivand et al. (2023) |
|  | ggpubr | 0.6.0 | Kassambara (2022) |
|  | gridGraphics | 0.5-1 | Murrell & Wen (2020) |
|  | glmmTMB | 1.1.9 | Brooks et al. (2017) |
|  | broom.mixed | 0.2.9.5 | Bolker & Robinson (2024) |
|  | zoo | 1.8-12 | Zeileis & Grothendieck (2005) |

Supplementary Table 2: Time periods from the CHELSA-TraCE21k dataset (Karger et al. 2020; Karger et al. 2023) used in this study. Further information can be found on the CHELSA webpage.

| **Time ID** | **Start year** | **End year** | **k-BP** |
| --- | --- | --- | --- |
| 10 | 900 | 999 | 1 |
| 0 | -100 | -1 | 2 |
| -10 | -1100 | -1001 | 3 |
| -20 | -2100 | -2001 | 4 |
| -30 | -3100 | -3001 | 5 |
| -40 | -4100 | -4001 | 6 |
| -50 | -5100 | -5001 | 7 |
| -60 | -6100 | -6001 | 8 |
| -70 | -7100 | -7001 | 9 |
| -80 | -8100 | -8001 | 10 |
| -90 | -9100 | -9001 | 11 |
| -100 | -10100 | -10001 | 12 |
| -110 | -11100 | -11001 | 13 |
| -120 | -12100 | -12001 | 14 |
| -130 | -13100 | -13001 | 15 |
| -140 | -14100 | -14001 | 16 |
| -150 | -15100 | -15001 | 17 |
| -160 | -16100 | -16001 | 18 |
| -180 | -18100 | -18001 | 20 |
| -190 | -19100 | -19001 | 21 |
| -200 | -20100 | -20001 | 22 |

## Supplementary Results

Supplementary Table 3: Mapping statistics of resequencing data.

| **Sample** | **Number of reads** | **Mapped reads** | **Properly paired reads** | **Genome-wide mean coverage** | **Insert size median (bp)** | **GC-content (%)** |
| --- | --- | --- | --- | --- | --- | --- |
| MF1 | 37512849 | 36680245 (97.78 %) | 33345658 (90.44 %) | 15.5912 | 297 | 32.61 |
| MF2 | 30925208 | 30305675 (98.00 %) | 27573498 (90.74 %) | 13.2659 | 298 | 32.15 |
| MF3 | 32731900 | 31546904 (96.38 %) | 28768950 (89.43 %) | 13.642 | 296 | 32.92 |
| MF4 | 32888448 | 32070040 (97.51 %) | 29402506 (90.99 %) | 16.6019 | 273 | 32.72 |
| MG2 | 29574915 | 29098966 (98.39 %) | 26464018 (91.07 %) | 12.4309 | 288 | 32.61 |
| MG3 | 29282686 | 28769324 (98.25 % ) | 26096752 (90.65 %) | 12.3216 | 298 | 32.63 |
| MG4 | 27560169 | 27107536 (98.36 %) | 24692740 (91.14 %) | 11.6459 | 289 | 32.55 |
| MG5 | 34723726 | 34098931 (98.20 %) | 31279922 (91.73 %) | 17.3902 | 272 | 33.45 |
| NMF1 | 39149237 | 38418718 (98.13 %) | 34846302 (90.58 %) | 19.2096 | 291 | 32.38 |
| NMF2 | 32848323 | 32332865 (98.43 %) | 29604680 (91.74 %) | 16.5641 | 279 | 33.07 |
| NMF3 | 29922750 | 29270955 (97.82 %) | 26781690 (91.13 %) | 15.2467 | 272 | 32.73 |
| NMF4 | 26926669 | 26460488 (98.27 %) | 24110414 (91.17 %) | 13.7049 | 277 | 32.71 |
| SI1 | 38628115 | 37887945 (98.08 %) | 34543616 (91.03 %) | 16.6717 | 294 | 32.58 |
| SI2 | 43087974 | 41349691 (95.97 %) | 37532316 (88.67 %) | 17.0428 | 288 | 32.68 |
| SI3 | 33478250 | 31731906 (94.78 %) | 28829146 (87.57 %) | 14.0101 | 300 | 32.67 |
| SI4 | 31364052 | 26697695 (85.12 %) | 24160270 (78.26 %) | 13.4785 | 287 | 32.23 |
| SS1 | 31635833 | 31148191 (98.46 %) | 28308742 (91.09 %) | 13.4556 | 298 | 32.62 |
| SS2 | 40525533 | 39859702 (98.36 %) | 36349244 (91.36 %) | 16.8263 | 289 | 31.91 |
| SS3 | 36668799 | 36066090 (98.36 %) | 32808088 (91.09 %) | 15.7296 | 288 | 31.94 |
| SS4 | 29302810 | 28821648 (98.36 %) | 26208916 (91.08 %) | 14.7396 | 285 | 33.12 |

Supplementary Table 4: Final diallelic SNP count per chromosome and individual of *Chironomus riparius.* Entries in mulithetsep files were used as input in the models of MSMC2.

| **Chromosome** | **Sample** | **SNP count** | **Entries in multihetsep** |
| --- | --- | --- | --- |
| 1 | MF1 | 902,195 | 183,734 |
| 1 | MF2 | 902,289 | 198,319 |
| 1 | MF3 | 901,883 | 129,659 |
| 1 | MF4 | 902,269 | 200,968 |
| 1 | MG2 | 902,062 | 218,160 |
| 1 | MG3 | 901,970 | 194,808 |
| 1 | MG4 | 902,245 | 232,985 |
| 1 | MG5 | 901,889 | 165,313 |
| 1 | NMF1 | 901,953 | 101,194 |
| 1 | NMF2 | 901,811 | 108,234 |
| 1 | NMF3 | 902,160 | 171,242 |
| 1 | NMF4 | 902,100 | 181,757 |
| 1 | SI1 | 902,294 | 204,944 |
| 1 | SI2 | 902,233 | 170,877 |
| 1 | SI3 | 902,079 | 195,934 |
| 1 | SI4 | 902,043 | 173,416 |
| 1 | SS1 | 902,274 | 225,600 |
| 1 | SS2 | 902,438 | 256,088 |
| 1 | SS3 | 902,255 | 223,891 |
| 1 | SS4 | 902,048 | 207,688 |
| 2 | MF1 | 913,108 | 228,105 |
| 2 | MF2 | 913,238 | 243,861 |
| 2 | MF3 | 912,982 | 221,618 |
| 2 | MF4 | 913,092 | 198,876 |
| 2 | MG2 | 913,102 | 245,326 |
| 2 | MG3 | 912,927 | 215,805 |
| 2 | MG4 | 912,954 | 239,476 |
| 2 | MG5 | 912,838 | 207,184 |
| 2 | NMF1 | 913,117 | 175,957 |
| 2 | NMF2 | 913,040 | 195,278 |
| 2 | NMF3 | 912,912 | 182,349 |
| 2 | NMF4 | 912,902 | 184,483 |
| 2 | SI1 | 913,081 | 219,691 |
| 2 | SI2 | 913,023 | 197,655 |
| 2 | SI3 | 912,842 | 157,342 |
| 2 | SI4 | 913,201 | 267,170 |
| 2 | SS1 | 912,946 | 213,785 |
| 2 | SS2 | 913,215 | 251,323 |
| 2 | SS3 | 913,209 | 258,693 |
| 2 | SS4 | 912,950 | 217,469 |
| 3 | MF1 | 786,653 | 102,172 |
| 3 | MF2 | 786,865 | 165,753 |
| 3 | MF3 | 786,770 | 176,347 |
| 3 | MF4 | 786,893 | 183,199 |
| 3 | MG2 | 786,842 | 192,167 |
| 3 | MG3 | 786,771 | 191,009 |
| 3 | MG4 | 786,804 | 148,405 |
| 3 | MG5 | 786,641 | 160,750 |
| 3 | NMF1 | 786,687 | 91,689 |
| 3 | NMF2 | 786,703 | 127,623 |
| 3 | NMF3 | 786,785 | 156,371 |
| 3 | NMF4 | 786,815 | 172,260 |
| 3 | SI1 | 787,059 | 217,880 |
| 3 | SI2 | 787,032 | 210,968 |
| 3 | SI3 | 786,868 | 196,400 |
| 3 | SI4 | 786,907 | 187,919 |
| 3 | SS1 | 786,861 | 191,661 |
| 3 | SS2 | 787,109 | 222,640 |
| 3 | SS3 | 786,921 | 212,354 |
| 3 | SS4 | 786,701 | 180,613 |
| 4 | MF1 | 246,169 | 45,355 |
| 4 | MF2 | 246,126 | 45,305 |
| 4 | MF3 | 246,121 | 40,092 |
| 4 | MF4 | 246,198 | 56,983 |
| 4 | MG2 | 246,135 | 46,197 |
| 4 | MG3 | 246,171 | 50,311 |
| 4 | MG4 | 246,137 | 53,441 |
| 4 | MG5 | 246,187 | 52,156 |
| 4 | NMF1 | 246,032 | 12,405 |
| 4 | NMF2 | 246,125 | 34,388 |
| 4 | NMF3 | 246,198 | 51,683 |
| 4 | NMF4 | 246,130 | 35,438 |
| 4 | SI1 | 246,229 | 53,298 |
| 4 | SI2 | 246,200 | 50,040 |
| 4 | SI3 | 246,234 | 57,178 |
| 4 | SI4 | 246,218 | 59,853 |
| 4 | SS1 | 246,184 | 52,714 |
| 4 | SS2 | 246,201 | 58,280 |
| 4 | SS3 | 246,175 | 50,133 |
| 4 | SS4 | 246,138 | 46,671 |

Supplementary Table 5: Summarising population specific parameters of *C. riparius* needed to convert recombination rates to estimate tMRCA in the MSMC2 analysis.

| **Population** | **Generations per year** (Oppold et al. 2016) | **Generation time** | **Recombination rate ρ (1/bp)** (Schmidt et al. 2020) | **Recombination rate r (cM/Mb)** | **Effective population size Ne**  (Oppold & Pfenninger 2017) |
| --- | --- | --- | --- | --- | --- |
| MG | 7.85 | 0.1274 | 0.068 | 1.37 | 3570000 |
| NMF | 7.7 | 0.1299 | 0.040 | 0.73 | 3950000 |
| MF | 9.07 | 0.1103 | 0.058 | 1.21 | 3450000 |
| SI | 10.57 | 0.0946 | 0.060 | 1.31 | 3300000 |
| SS | 14.86 | 0.0673 | 0.066 | 2.20 | 2150000 |
| Mean | 10.01 | 0.0999 |  | 1.36 |  |

Supplementary Table 6: Summary of the GLMM analysis with beta distribution and the BFGS algorithm for model optimization (glmmTMB(rel.cc ~ temperature * time + (1 | Population), data = data, family = beta_family(), control = glmmTMBControl(optimizer = optim, optArgs = list(method = "BFGS")), dispformula = ~time). The effect of temperature, time, and their interaction on the response variable (relative cross-coalescence rate) are evaluated, while accounting for random intercepts by population. Fixed effects estimates, standard errors, z-values, p-values, and significance levels are shown. The dispersion model is specified as a function of time. Model fit statistics, including AIC, BIC, log likelihood, and deviance, are also reported.

| **Term** | **Estimate** | **Std. Error** | **z Value** | **Pr(>z)** | **Significance** |
| --- | --- | --- | --- | --- | --- |
| **Fixed Effects** |  |  |  |  |  |
| (Intercept) | -1.935 | 0.203 | -9.54 | < 2e-16 | *** |
| Temperature | 0.0003 | 0.0159 | 0.019 | 0.985 |  |
| Time | 0.000167 | 0.00001 | 16.929 | < 2e-16 | *** |
| Temperature:Time | 0.000002 | 0.00000078 | 2.758 | 0.00581 | ** |
| **Random Effects** |  |  |  |  |  |
| Population (Intercept) | Variance = 0.0215 | Std. Dev. =  0.1467 | |  |  |
| **Dispersion Model** |  |  |  |  |  |
| (Intercept) | 3.155 | 0.121 | 26.145 | < 2e-16 | *** |
| Time | 0.000042 | 0.000009 | 4.905 | 9.32E-07 | *** |
|  |  |  |  |  |  |
| **Model Fit Information** |  |  |  |  |  |
| AIC | -1106.9 |  |  |  |  |
| BIC | -1078.3 |  |  |  |  |
| Log Likelihood | 560.5 |  |  |  |  |
| Deviance | -1120.9 |  |  |  |  |
| Number of Observations | 440 |  |  |  |  |
| Number of Groups (Population) | 5 |  |  |  |  |
|  |  |  |  |  |  |
| Significance Codes | ***: p < 0.001, **: p < 0.01, *: p < 0.05, .: p < 0.1, '' : p ≥ 0.1 | | | | |

Supplementary Table 7: Centromere predictions based on RepeatOBserver (Elphinstone et al. 2025).

| **Chromosome** | **Length (bp)** | **Start (bp)** | **End (bp)** |
| --- | --- | --- | --- |
| Chr1 | 11,885,000 | 24,007,501 | 35,892,501 |
| Chr2 | 8,970,000 | 27,232,501 | 36,202,501 |
| Chr3 | 6,860,000 | 20,902,501 | 27,762,501 |
| Chr4 | 1,850,000 | 7,722,501 | 9,572,501 |

Supplementary Figure 1: MSMC2 analysis visualizing generations reaching into the past.


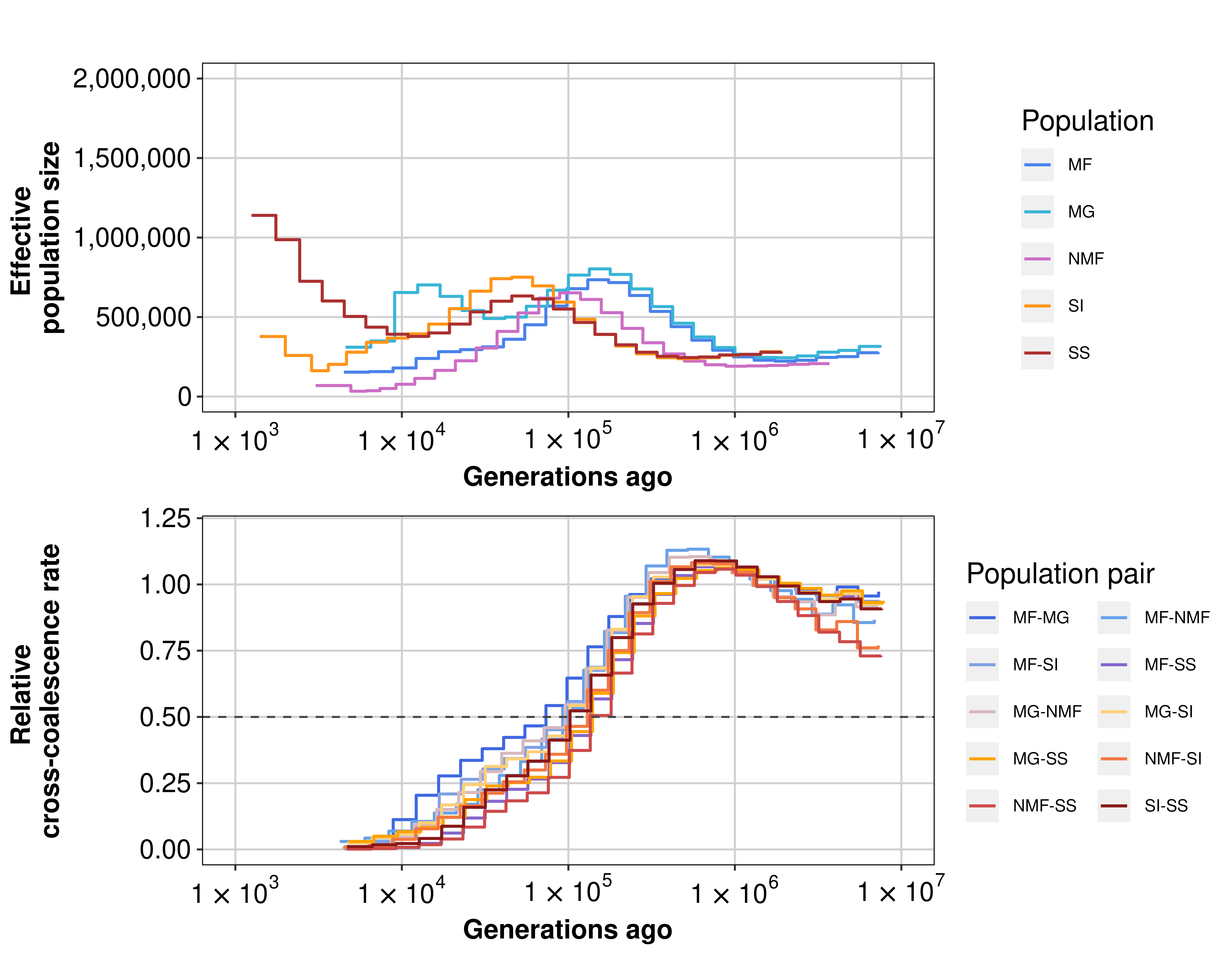


Supplementary Figure 2: Residuals of GLMM.


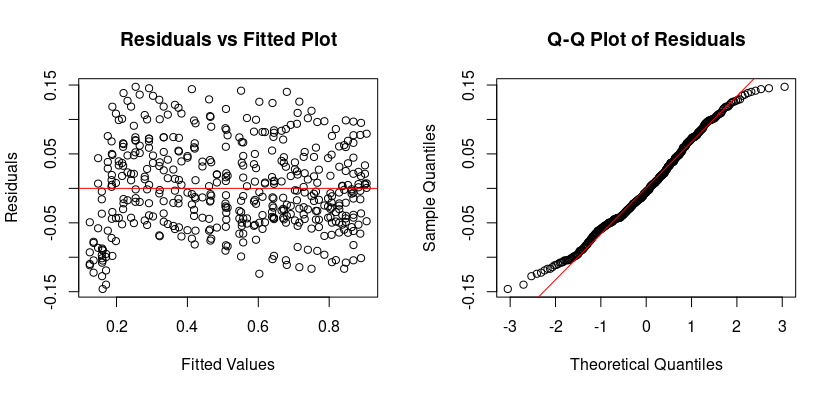


Supplementary Figure 3: Summary plot of D-GENIES (Cabanettes and Klopp 2018).


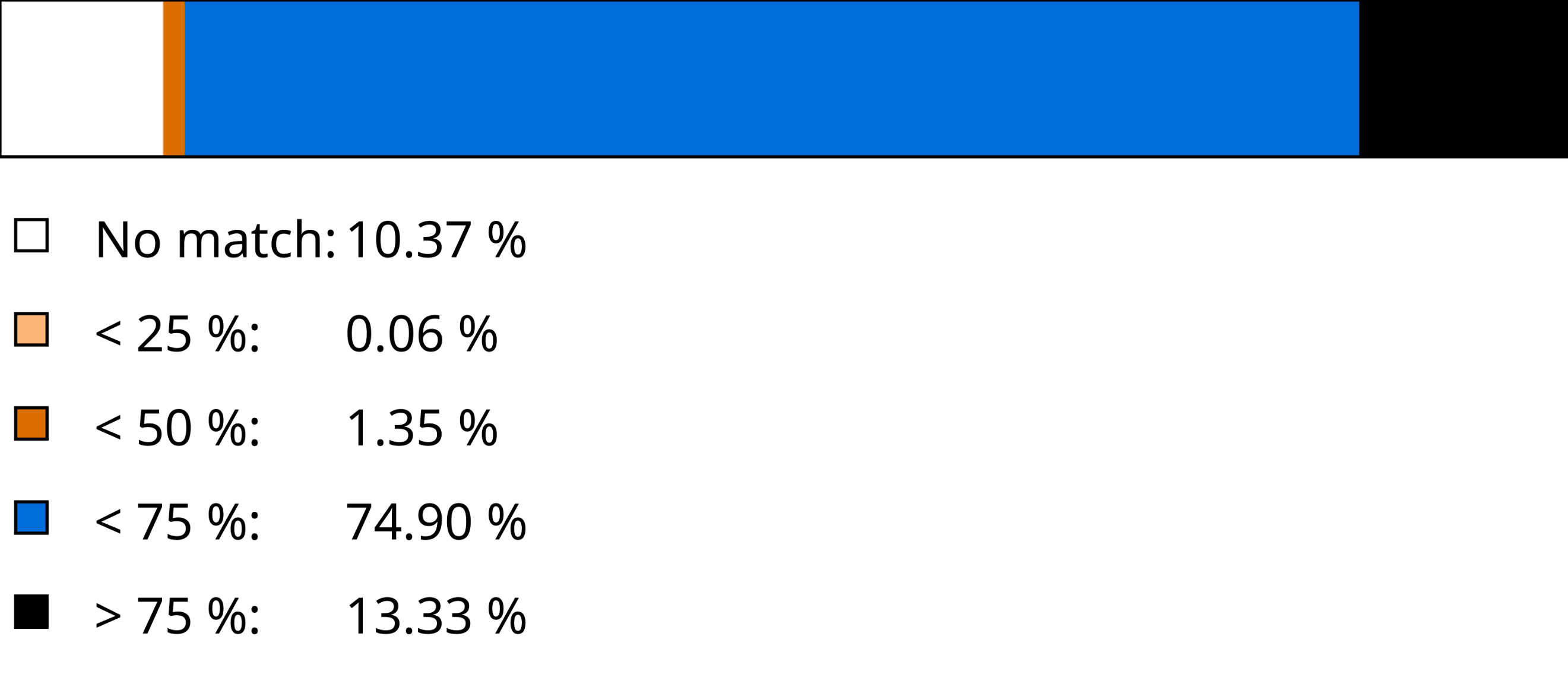


## References

Andrews S. 2010. FastQC: A Quality Control Tool for High Throughput Sequence Data. Babraham Bioinformatics. http://www.bioinformatics.babraham.ac.uk/projects/fastqc/.

Auguie B. 2017. gridExtra: Miscellaneous Functions for “Grid” Graphics. R package version 23. https://CRAN.R-project.org/package=gridExtra.

Auguie B. 2019. egg: Extensions for “ggplot2”: Custom Geom, Custom Themes, Plot Alignment, Labelled Panels, Symmetric Scales, and Fixed Panel Size. https://cran.r-project.org/package=egg.

Bivand R, Keitt T, Rowlingson B. 2023. rgdal: Bindings for the “Geospatial” Data Abstraction Library. https://CRAN.R-project.org/package=rgdal.

Bivand R, Lewin-Koh N. 2023. maptools: Tools for Handling Spatial Objects. https://CRAN.R-project.org/package=maptools.

Bivand R, Rundel C. 2023. rgeos: Interface to Geometry Engine - Open Source ('GEOS’). https://CRAN.R-project.org/package=rgeos.

Bolger AM, Lohse M, Usadel B. 2014. Trimmomatic: a flexible trimmer for Illumina sequence data. Bioinformatics. 30(15):2114–2120. doi:10.1093/BIOINFORMATICS/BTU170. https://academic.oup.com/bioinformatics/article/30/15/2114/2390096.

Bolker B, Robinson D. 2024. broom.mixed: Tidying Methods for Mixed Models. https://CRAN.R-project.org/package=broom.mixed.

Broad Institute. 2018. Picard Tools. http://broadinstitute.github.io/picard/.

Brooks ME, Kristensen K, van Benthem KJ, Magnusson A, Berg CW, Nielsen A, Skaug HJ, Maechler M, Bolker BM. 2017. glmmTMB Balances Speed and Flexibility Among Packages for Zero-inflated Generalized Linear Mixed Modeling. R J. 9(2):378–400. doi:10.32614/RJ-2017-066.

Cabanettes F, Klopp C. 2018. D-GENIES: Dot plot large genomes in an interactive, efficient and simple way. PeerJ. 2018(6):e4958. doi:10.7717/PEERJ.4958/TABLE-2.

Camacho C, Coulouris G, Avagyan V, Ma N, Papadopoulos J, Bealer K, Madden TL. 2009. BLAST+: Architecture and applications. BMC Bioinformatics. 10(1):1–9. doi:10.1186/1471-2105-10-421.

Challis R, Richards E, Rajan J, Cochrane G, Blaxter M. 2020. BlobToolKit – Interactive Quality Assessment of Genome Assemblies. G3 Genes|Genomes|Genetics. 10(4):1361–1374. doi:10.1534/G3.119.400908.

Delaneau O, Zagury JF, Robinson MR, Marchini JL, Dermitzakis ET. 2019. Accurate, scalable and integrative haplotype estimation. Nature Communications 2019 10:1. 10(1):1–10. doi:10.1038/s41467-019-13225-y.

Elphinstone C, Elphinstone R, Todesco M, Rieseberg LH. 2025. RepeatOBserver: Tandem Repeat Visualisation and Putative Centromere Detection. Mol Ecol Resour.:e14084. doi:10.1111/1755-0998.14084.

Ewels P, Magnusson M, Lundin S, Käller M. 2016. MultiQC: Summarize analysis results for multiple tools and samples in a single report. Bioinformatics. 32(19):3047–3048. doi:10.1093/bioinformatics/btw354.

Hijmans RJ, van Etten J. 2012. raster: Geographic analysis and modeling with raster data. http://cran.r-project.org/package=raster.

Karger DN, Nobis MP, Normand S, Graham CH, Zimmermann NE. 2020. CHELSA-TraCE21k: Downscaled transient temperature and precipitation data since the last glacial maximum. EnviDat. doi:10.16904/envidat.211.

Karger DN, Nobis MP, Normand S, Graham CH, Zimmermann NE. 2023. CHELSA-TraCE21k – high-resolution (1km) downscaled transient temperature and precipitation data since the Last Glacial Maximum. Climate of the Past. 19(2):439–456. doi:10.5194/cp-19-439-2023.

Kassambara A. 2022. ggpubr: “ggplot2” Based Publication Ready Plots. https://CRAN.R-project.org/package=ggpubr.

Li H. 2009. SNPable Regions. http://lh3lh3.users.sourceforge.net/snpable.shtml.

Li H. 2013. Aligning sequence reads, clone sequences and assembly contigs with BWA-MEM. [accessed 2021 Feb 6]. http://arxiv.org/abs/1303.3997.

Li H, Handsaker B, Wysoker A, Fennell T, Ruan J, Homer N, Marth G, Abecasis G, Durbin R, Subgroup 1000 Genome Project Data Processing. 2009. The Sequence Alignment/Map format and SAMtools. Bioinformatics. 25(16):2078–2079. doi:10.1093/bioinformatics/btp352. https://doi.org/10.1093/bioinformatics/btp352.

Manni M, Berkeley MR, Seppey M, Zdobnov EM. 2021. BUSCO: Assessing Genomic Data Quality and Beyond. Curr Protoc. 1(12):e323. doi:10.1002/CPZ1.323.

Massicotte P, South A. 2023. rnaturalearth: World Map Data from Natural Earth. https://CRAN.R-project.org/package=rnaturalearth.

Murrell P, Wen Z. 2020. gridGraphics: Redraw Base Graphics Using “grid” Graphics. https://CRAN.R-project.org/package=gridGraphics.

Neuwirth E. 2022. RColorBrewer: ColorBrewer Palettes. R package version 11-3. [accessed 2024 Mar 18]. https://CRAN.R-project.org/package=RColorBrewer.

Okonechnikov K, Conesa A, García-Alcalde F. 2016. Qualimap 2: advanced multi-sample quality control for high-throughput sequencing data. Bioinformatics. 32(2):292–294. doi:10.1093/bioinformatics/btv566.

Oppold AM, Pedrosa JAM, Bálint M, Diogo JB, Ilkova J, Pestana JLT, Pfenninger M. 2016. Support for the evolutionary speed hypothesis from intraspecific population genetic data in the non-biting midge *Chironomus riparius*. Proceedings of the Royal Society B: Biological Sciences. 283(1825). doi:10.1098/RSPB.2015.2413.

Oppold AM, Pfenninger M. 2017. Direct estimation of the spontaneous mutation rate by short-term mutation accumulation lines in *Chironomus riparius*. Evol Lett. 1(2):86–92. doi:10.1002/EVL3.8.

Pebesma E. 2018. Simple Features for R: Standardized Support for Spatial  Vector Data. R J. 10(1):439–446. doi:10.32614/RJ-2018-009.

Pebesma E, Bivand R. 2023. Spatial Data Science: With applications in R. Chapman and Hall/CRC.

Pedersen TL. 2024. patchwork: The Composer of Plots. https://patchwork.data-imaginist.com.

Quinlan AR, Hall IM. 2010. BEDTools: a flexible suite of utilities for comparing genomic features. Bioinformatics. 26(6):841–842. doi:10.1093/BIOINFORMATICS/BTQ033.

R Core Team. 2020. R: A language and environment for statistical computing. https://www.R-project.org/.

RStudio Team. 2020. RStudio: Integrated Development Environment for R. https://www.rstudio.com/.

Schiffels S. 2021 Sep 7. Tools and Utilities for msmc and msmc2. https://github.com/stschiff/msmc-tools.

Schiffels S, Wang K. 2020. MSMC and MSMC2: The Multiple Sequentially Markovian Coalescent. Methods in Molecular Biology. 2090:147–166. doi:10.1007/978-1-0716-0199-0_7.

Schmidt H, Hellmann SL, Waldvogel AM, Feldmeyer B, Hankeln T, Pfenninger M. 2020. A High-Quality Genome Assembly from Short and Long Reads for the Non-biting Midge *Chironomus riparius* (Diptera). G3 Genes|Genomes|Genetics. 10(4):1151–1157. doi:10.1534/G3.119.400710.

Simão FA, Waterhouse RM, Ioannidis P, Kriventseva E V., Zdobnov EM. 2015. BUSCO: assessing genome assembly and annotation completeness with single-copy orthologs. Bioinformatics. 31(19):3210–3212. doi:10.1093/BIOINFORMATICS/BTV351.

Smit AFA, Hubley R, Grenn P. 2015. RepeatMasker Open-4.0. http://www.repeatmasker.org.

South A. 2017. rnaturalearthdata: World Vector Map Data from Natural Earth Used in “rnaturalearth.” https://CRAN.R-project.org/package=rnaturalearthdata.

Tange O. 2011. GNU Parallel - The Command-Line Power Tool. login: The USENIX Magazine. 36(1):42–47. doi:10.5281/zenodo.16303. http://www.gnu.org/s/parallel.

Wickham H. 2007. Reshaping Data with the reshape Package. J Stat Softw. 21(12):1–20. doi:10.18637/JSS.V021.I12.

Wickham H. 2016. ggplot2: Elegant Graphics for Data Analysis. https://ggplot2.tidyverse.org.

Wickham H, Averick M, Bryan J, Chang W, D’ L, Mcgowan A, François R, Grolemund G, Hayes A, Henry L, et al. 2019. Welcome to the Tidyverse. J Open Source Softw. 4(43):1686. doi:10.21105/JOSS.01686.

Wickham H, François R, Henry L, Müller K, Vaughan D. 2023. dplyr: A Grammar of Data Manipulation.

Wickham H, Seidel D. 2022. scales: Scale Functions for Visualization. R package version 121. https://CRAN.R-project.org/package=scales.

Wilke CO. 2024. cowplot: Streamlined Plot Theme and Plot Annotations for “ggplot2.” https://CRAN.R-project.org/package=cowplot.

Zeileis A, Grothendieck G. 2005. zoo: S3 Infrastructure for Regular and Irregular Time Series. J Stat Softw. 14(6):1–27. doi:10.18637/jss.v014.i06.
